# *cxcl12a* plays an essential role in pharyngeal cartilage development

**DOI:** 10.3389/fcell.2023.1243265

**Published:** 2023-10-04

**Authors:** Zhaohui Wei, Qiang Hong, Zijiao Ding, Jingwen Liu

**Affiliations:** ^1^ School of Basic Medicine, Anhui Medical University, Hefei, Anhui, China; ^2^ College of Veterinary Medicine, Yunnan Agricultural University, Kunming, Yunnan, China

**Keywords:** *Cxcl12a*, chemokine signal, pharyngeal arches, craniofacial cartilage, neural crest cells

## Abstract

**Background:** Neural crest cells constitute a distinct set of multipotent cells that undergo migration along predefined pathways, culmination in the differentiation into a plethora of cell types, including components of the pharyngeal cartilage. The neurocranium is composite structure derived from both cranial neural crest and mesoderm cells, whereas the pharyngeal skeletal elements-including the mandibular and branchial arches-are exclusively formed by craniofacial neural crest cells. Previous studies have elucidated the critical involvement of the chemokine signaling axis Cxcl12b/Cxcr4a in craniofacial development in zebrafish (*Danio rerio*). Nonetheless, the function contribution of Cxcl12a and Cxcr4b-the homologous counterparts of Cxcl12b and Cxcr4a-remain largely unexplored.

**Methods:** In the present study, mutant lines for *cxcl12a* and *cxcr4b* were generated employing CRISPR/Cas9 system. Temporal and spatial expression patterns of specific genes were assessed using *in situ* hybridization and dual-color fluorescence *in situ* hybridization techniques. High-resolution confocal microscopy was utilized for *in vivo* imaging to detect the pharyngeal arch or pouch patterning. Additionally, cartilage formation within the craniofacial region was analyzed via Alcian blue staining, and the proliferation and apoptosis rates of craniofacial neural crest cells were quantified through BrdU incorporation and TUNEL staining.

**Results:** Our data reveals that the deletion of the chemokine gene *cxcl12a* results in a marked diminution of pharyngeal cartilage elements, attributable to compromised proliferation of post-migratory craniofacial neural crest cells. Subsequent experiments confirmed that Cxcl12a and Cxcl12b exhibit a synergistic influence on pharyngeal arch and pouch formation.

**Conclusion:** Collectively, the present investigation furnishes compelling empirical evidence supporting the indispensable role of Cxcl2a in craniofacial cartilage morphogenesis, albeit *cxcr4b* mutants exert a minimal impact on this biological process. We delineate that Cxcl12a is essential for chondrogenesis in zebrafish, primarily by promoting the proliferation of craniofacial neural crest cells. Furthermore, we proposed a conceptual framework wherein Cxcl12a and Cxcl12b function synergistically in orchestrating both the pharyngeal arch and pouch morphogenesis.

## 1 Introduction

The craniofacial skeletal framework constitutes a pivotal element in vertebrate morphogenesis, serving indispensable functions in the encapsulation and protection of the brain, as well as facilitating vital processes such as respiration, alimentary intake, and communicative functions. Predominantly, the craniofacial bones emanate from cranial neural crest cells (CNCCs), a specialized subpopulation of neural crest cells (NCCs) with multipotent characteristics (LaBonne and Bronner-Fraser, 1998). These NCCs exhibit remarkable migratory capabilities and differentiate into a broad spectrum of cellular lineages, including the components of the pharyngeal skeleton. Notably, the developmental dynamics of CNCCs have attracted considerable scholarly attention, largely attributable to the plethora of congenital human maladies, such as Waardenburg’s syndrome (Waardenburg, 1951), Treacher Collins syndrome (Sulik et al., 1987) and Pierre-Robin syndrome (Dennison, 1965). These conditions are manifest as aberrant craniofacial phenotypes and arise from perturbations in CNCC migration and pharyngeal arch development. In orchestrating these intricate developmental processes, CNCCs interact with a complex molecular milieu, involving signaling pathways such as Sonic Hedgehog (Shh) (Wada et al., 2005; Eberhart et al., 2006), Fibroblast Growth Factor (FGF) (Crump et al., 2004), and Retinoic Acid (RA) (Kopinke et al., 2006). Although recent years have witnessed substantial advancements in our understanding of NCC ontogeny and pharyngeal cartilage morphogenesis, the precise molecular mechanisms underlying these processes remain incompletely elucidated.

Chemokines, small proteins with chemoattractant properties, represent another layer of complexity in vertebrate development and are classified into four subgroups based on the spatial arrangement of cysteine residues (C, CC, CXC, and CX3C) (Raz and Mahabaleshwar, 2009). Particularly, the stromal cell-derived factor 1 (SDF-1, also known as CXCL12), a member of the CXC subfamily, and its cognate receptor CXCR4, have garnered interest for their roles in modulating cellular proliferation and adhesion, and migration during embryogenesis (Peled et al., 1999; Raz and Mahabaleshwar, 2009; Boldajipour et al., 2011; Liu et al., 2019). Earlier reports have ascertained that Cxcl12 exhibits specific expression in the pharyngeal endoderm concomitant with CNCC migration in murine and avian models, while its receptor-Cxcr4 is expressed in the pharyngeal NCCs (Escot et al., 2016). Disruptions in the CXCR4 signaling axis impair CNCC migration into the pharyngeal arches, culminating in congenital malformations of the lower jaw and hyoid bone (Escot et al., 2016). Given the genomic duplications in *Danio rerio* (zebrafish), two homologous CXCL12 ligands (Cxcl12a and Cxcl12b) and two CXCR4 receptors (Cxcr4a and Cxcr4b) are present, each with distinct expression patterns and functional roles in cellular cell adhesion and migration (Peled et al., 1999; Colak-Champollion et al., 2019). The Cxcl12a-Cxcr4b axis has been implicated in a myriad of physiological processes, including but not limited to the primordial germ cell and lateral line primordium migration, the guidance of truck lymphatic network (David et al., 2002; Doitsidou et al., 2002; Knaut et al., 2003). Conversely, the Cxcl12b-Cxcr4a signaling axis has been predominantly studied for its role in endodermal migration and vascular patterning, with particular emphasis on craniofacial development (Nair and Schilling, 2008; Olesnicky Killian et al., 2009; Siekmann et al., 2009; Li et al., 2018).

In light of the demonstrated impact of the Cxcl12b-Cxcr4a signaling axis on craniofacial morphogenesis, the present study aims to elucidate the functional significance of their homologous counterparts, Cxcl12a and Cxcr4b, in this intricate developmental process. Utilizing *D. rerio* (zebrafish) as a model organism, we meticulously investigated the role of Cxcl12a in orchestrating craniofacial development. Initially, the spatiotemporal expression profiles of *cxcl12a* and *cxcr4b* were ascertained within the developing pharyngeal region. Leveraging *in vivo* gene manipulation techniques, namely, knock-down and knock-out strategies, we discerned that Cxcl12a is indispensable for the genesis of craniofacial cartilage—an effect that could be substantially ameliorated via the exogenous administration of *cxcl12a* mRNA. Intriguingly, the observed cartilaginous defects in *cxcl12a* mutant lines emanated from compromised proliferation of cranial neural crest cells (CNCCs) subsequent to their migratory phase. Furthermore, our results unveil a synergistic functionality between *cxcl12a* and *cxcl12b* in the formation of pharyngeal arches and pouches. Collectively, our findings delineate the crucial role of Cxcl12a in craniofacial cartilage development and accentuate the integral contributions of chemokine signaling networks to the ontogenesis of the pharyngeal apparatus.

## 2 Materials and methods

### 2.1 Zebrafish husbandry and strains

The wild-type embryos employed in the present study were procured through natural mating of the AB strain zebrafish. Post-fertilization, embryos were incubated in a Holtfreter’s solution at an ambient temperature of 28.5°C. Embryonic staging was conducted according to discernible morphological landmarks. For the purpose of visualizing pharyngeal pouch cells, transgenic lines *Tg(sox17:GFP)* and *Tg(nkx2.3-mCherry)* were utilized. *Tg(sox10:GFP; nkx2.3-mCherry)* and *Tg(fli1:GFP; nkx2.3-mCherry)* lines were employed to delineate both the pharyngeal arch cells and pouch cells.

### 2.2 Zebrafish *cxcl12a* and *cxcr4b* mutant line generation

Utilizing the CRISPR/Cas9 genome-editing technique, single guide RNAs (sgRNAs) were engineered to target exon 1 of *cxcl12a* and exon 2 of *cxcr4b*. The sequence corresponding to the sgRNA target in *cxcl12a* exon 1 was: 5’-GGT​CGC​CAT​TCA​TGC​ACC​GAT​TTC​CAA-3’. For *cxcr4b* exon 2, the sequence was: 5’-CTT​ACT​GTG​CCG​GCA​TCC​GG-3’. Subsequent to the CRISPR/Cas9-mediated manipulation, frameshift mutations were observed—specifically, a four-base-pair deletion in *cxcl12a* and a thirteen-base-pair deletion in *cxcr4b*. These deletions resulted in premature stop codons in the respective mRNAs. Homozygous mutants of *cxcl12a* ^−/−^ and *cxcr4b* ^−/−^ were isolated through the screening of progeny from heterozygous mutant parents.

### 2.3 Microinjection

For *cxcl12a* and *cxcl12b*, capped messenger RNAs (mRNAs) were synthesized *in vitro* from the linearized plasmid templates employing the mMessage mMachine kit (Ambion). Control morpholinos, constituting the sequence (5’-CCT​CTT​ACC​TCA​GTT​ACA​ATT​TAT​A-3’), were obtained along with ATG morpholinos (MOs) specifically targeting *cxcl12b* (5'-CGC​TAC​TAC​TTT​GCT​ATC​CAT​GCC​A-3') and MO targeting the start codon of *cxcl12a* (5'-ATC​ACT​TTG​AGA​TCC​ATG​TTT​GCA-3'). These were procured from Gene Tools (Philomath, OR, United States), were microinjected in accordance with previously established protocols (Dambly-Chaudiere et al., 2007; Hollway et al., 2007; Nair and Schilling, 2008).

### 2.4 Whole-mount *in situ* hybridization

Whole-mount *in situ* hybridization assays were executed in strict adherence to previously published protocols (Ning et al., 2013). In dual-color fluorescence *in situ* hybridization, anti-Digoxigenin-POD (11633716001, Roche) and anti-Fluorescein-POD (11426346910, Roche) were deployed as the primary antibodies to discern Digoxigenin-labeled mCherry probes and Fluorescein-labeled *cxcl12a* or *cxcr4b* probes, respectively. Subsequent analysis was performed utilizing the Perkin Elmer TAS fluorescein system (NEL701A001KT) as delineated in earlier publications (Mao et al., 2019). The composite *in situ* hybridization images display the following annotations: upper left quadrant designates the genotype, upper right indicates developmental stage, lower left specifies the probe in use, and the lower right reveals the aggregate count of representative samples alongside detected samples.

### 2.5 Reverse-transcription quantitative polymerase chain reaction (RT-qPCR)

Transcript levels of *cxcl12a*, *cxcr4b*, *hand2*, and *sox10* were ascertained from a composite pool of 30 embryos at specified developmental intervals. Total RNA was extracted employing TRIzol reagent (Invitrogen, Beijing, China) and purified to an A260/A280 optical density ratio of 2.0. Subsequently, 2 µg of each sample was subjected to reverse transcription utilizing 5X RT MasterMix (Abcam, Beijing, China, G592). Quantitative PCR assays were conducted employing TB Green Premix Ex Taq II (Takara, RR820A) within an Mx3000P real-time PCR system (Stratagene). Expression levels of the genes under investigation were quantified via the 2^−ΔΔCT^ method and were normalized relative to β-actin transcripts. The fold-change in mRNA expression is presented in relation to wild-type embryo controls.

### 2.6 Confocal imaging

Zebrafish embryos were anaesthetized and positioned in a 2% low melting point agarose matrix (0815, AMRESCO) immersed in egg water. Temporally specific live imaging was executed employing a Nikon A1R^+^ confocal microscope. All resultant confocal images were processed and analyzed utilizing Nikon NIS-Elements AR 4.13.00 software.

### 2.7 Alcian blue staining

Embryos were initially fixed in a 4% paraformaldehyde solution overnight and subsequently rinsed with a 0.1% Tween-20 infused distilled water until transparency was achieved. The specimens were then stained with an Alcian blue buffer solution (comprising 0.015% Alcian Blue, 80% ethanol, and 20% acetic acid) overnight at ambient temperature. A destaining process ensued, utilizing ethanol as the solvent. Subsequently, the embryos were enzymatically treated with a 0.5% trypsin solution (0458, AMRESCO) in a supersaturated borax medium until the specimens attained a softer consistency. Post-trypsin treatment, embryos were incubated in a 1% KOH solution for 30 min, followed by rinsing in 0.1% Tween-20 infused water. Lastly, embryos were subjected to a gradient of glycerol concentrations and subsequently imaged via microscopy.

### 2.8 Terminal deoxynucleotidyl transferase dUTP nick end labeling (TUNEL)

The *In Situ* Cell Death Detection Kit, TMR Red (Roche, 12156792910), was employed for the TUNEL assays in accordance with the manufacturer’s guidelines. Embryos harvested at specified stages were fixed in 4% paraformaldehyde at 4°C overnight, followed by a triple wash in PBST and proteinase K-mediated permeabilization. The permeabilized embryos were subsequently re-fixed in 4% paraformaldehyde for 30 min at room temperature, washed thrice in PBST, and incubated overnight at 4°C in a TUNEL-labeling milieu. Following triple PBST washes, the embryos were mounted in 2% low-melting-point agarose and visualized through confocal microscopy.

### 2.9 Proliferation analyses

For BrdU incorporation assays, embryos were exposed to a 10 mM BrdU solution at designated stages for a duration of 20 min and were subsequently harvested. Incorporated BrdU and GFP were detected using anti-BrdU antibodies (1:1,000; B5002, Sigma) and rabbit anti-GFP antibodies (1:1,000, ab183734, Abcam) via whole-mount immunostaining.

### 2.10 Statistical analysis

Data were subjected to analytical scrutiny utilizing GraphPad Prism 9 software and are articulated as the mean ± standard deviation (SD). Comparisons between control and experimental cohorts were conducted through an unpaired, two-tailed Student’s *t*-test. Levels of statistical significance were demarcated as follows: *p* < 0.05 was considered statistically significant and designated with a single asterisk (*), *p* < 0.01 was deemed highly significant and marked with double asterisks (**), and *p* < 0.001 was categorized as exceedingly significant and annotated with triple asterisks (***).

## 3 Results

### 3.1 *cxcl12a* has specific expression in pharyngeal pouch

**FIGURE 1:**
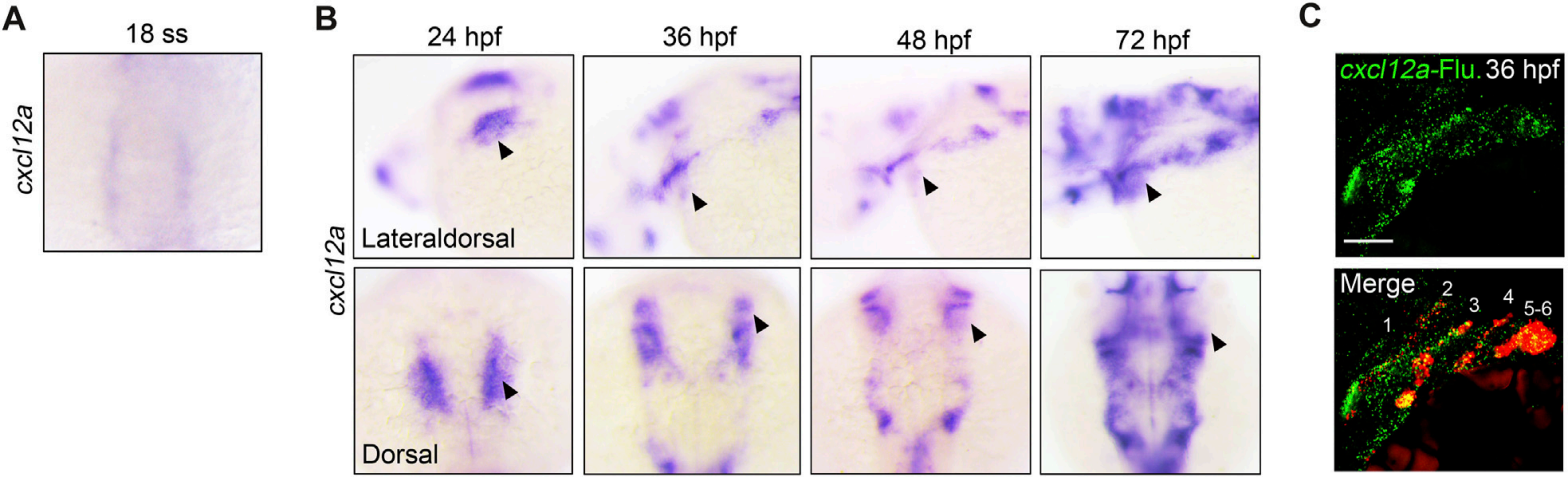
Expression of *cxcl12a* in endodermal pouches. **(A)** Temporal evaluation of *cxcl12a* transcriptional activity at 18-somite stage. **(B)** Spatiotemporal dynamics of *cxcl12a* expression within the developmental window ranging from 24 hpf to 72 hpf. Triangular markers delineate regions manifesting *cxcl12a* transcripts in the pharyngeal area. **(C)** Concomitant fluorescence *in situ* hybridization of *cxcl12a* at 36 hpf employing *Tg(nkx2.3:mCherry)* transgenic embryos. Probes used for staining include *cxcl12a*-Fluorescence probe (green) and mCherry-DNP (red) probes. Scale bar, 50 μm.

Previous investigations have elucidated the pivotal role of *cxcl12b* in cranial neural crest cell (CNCC) migration and neurocranium morphogenesis, originating from the pharyngeal endoderm and arch mesenchyme (Olesnicky Killian et al., 2009). Additionally, *dmrt2b* has been identified as an upstream regulator of *cxcl12b,* thereby influencing pharyngeal neural crest cell ontogenesis (Li et al., 2018). In light of these findings, our study aims to interrogate the influence of *cxcl12a* and its cognate receptor, *cxcr4b—*homologous counterparts to *cxcl12b* and *cxcr4a* in zebrafish—on craniofacial development. An initial examination of the spatiotemporal expression patterns of *cxcl12a* and *cxcr4b* was conducted employing whole-mount *in situ* hybridization (WISH) experiments at distinct developmental stages. Intriguingly, while no expression of *cxcl12a* was discerned in CNCCs within the timeframe of 14–17 h post-fertilization (hpf) (from ZFIN. org), specific expression was observed in the pharyngeal region commencing at 18 hpf, coinciding with the manifestation of the first pharyngeal pouch (Figure 1A). During subsequent developmental intervals, ranging 24 hpf to 72 hpf, there was a conspicuous enrichment of *cxcl12a* expression in the pharyngeal sector (Figure 1B). To further delineate the precise anatomical locus of *cxcl12a* expression within the pharyngeal domain, dual-color fluorescence *in situ* hybridization assays were deployed to examine the exact expression of *cxcl12a* in the *Tg (nkx2.3: mCherry)* transgenic embryos. The outcomes, illustrated in Figure 1C unequivocally established co-localization of *cxcl12a* transcripts with mCherry-tagged pharyngeal pouch cells at 36 hpf. In contrast, *cxcr4b* exhibited expression within the pharyngeal region but was conspicuously absent in the pharyngeal pouches (Supplementary Figures S1A–C). Collectively, these data cogently substantiate that *cxcl12a* is distinctly localized within the pharyngeal pouches.

### 3.2 Loss of *cxcl12a* leads to pharyngeal cartilage malformation

In pursuit of further delineating the contributory roles of *cxcl12a* and *cxcr4b* in craniofacial ontogenesis, targeted mutations in both genes were engendered via the CRISPR/Cas9 genomic editing system (Supplementary Figures S2A, B). Notably, aberrations in *nanos3* localization, a marker for the primordial germ cells, were discernibly manifested in homozygous *cxcl12a* and *cxcr4b* mutants (Supplementary Figure S2C) (Boldajipour et al., 2008). Subsequent evaluations employing whole-mount *in situ* hybridization (WISH) and quantitative real-time polymerase chain reaction (qRT-PCR) revealed precipitous decrements in the expression levels of both *cxcl12a* and *cxcr4b* within these mutants (Supplementary Figures S2D, E). These observations rigorously authenticate these mutants as *bona fide* loss-of-function strains.

**FIGURE 2:**
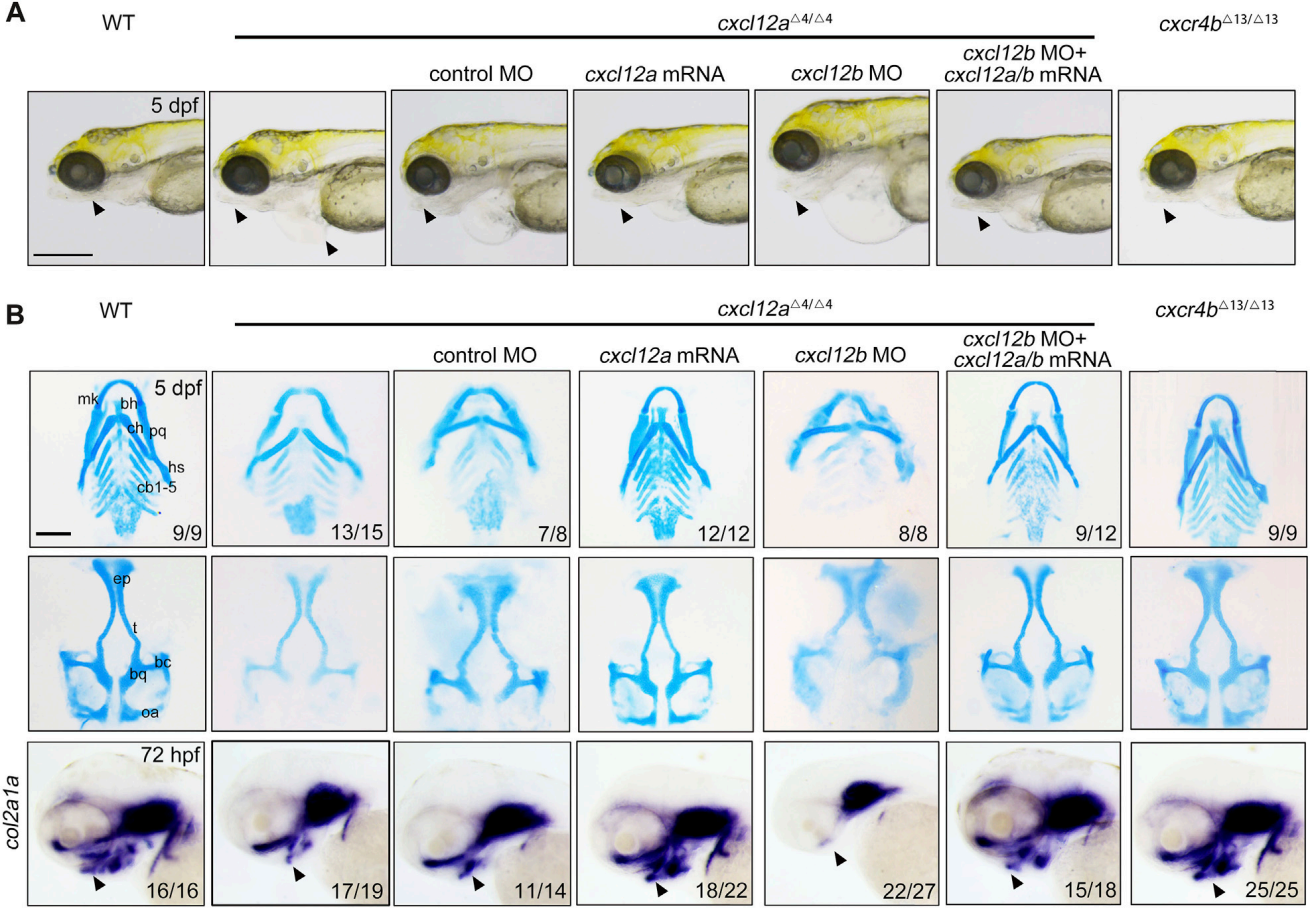
Depletion of cxcl12a impairs craniofacial cartilage development. **(A)** Phenotypic assessment of embryos at designated developmental stages, noting discernible craniofacial aberrations and pericardial edema in *cxcl12a* mutant embryos. The triangle annotates the zebrafish mandibular region. Scale bars set at 200 mm. **(B)** Alcian blue histochemical staining of craniofacial cartilage structures at 5 dpf and corresponding *in situ* hybridization images of *col2a1a*. Anatomical components denoted are: ep, ethmoid plate; tc, trabeculae cranii; mk, Meckel’s cartilage; bh, basihyal; ch, ceratohyal; pq, palatoquadrate; hs, hyosymplectic; cb, ceratobranchial. The triangle designates the mandibular sector. Scale bars: 100 µm.

A comprehensive phenotypic scrutiny revealed that *cxcl12a* mutants exhibited symptoms of pericardial edema coupled with mandibular hypoplasia at 5 days post-fertilization (dpf), anomalies amenable to amelioration via *cxcl12a* mRNA microinjection (Figure 2A). In contrast, *cxcr4b* ablation failed to induce any overt morphological perturbations in the resultant mutants (Figure 2A). Alcian blue histochemical analyses revealed a substantive attenuation in the structural integrity of both Meckel’s cartilage and palatoquadrate, as well as a marked diminution in the number of ceratobranchial cartilages in *cxcl12a* mutants, thereby implicating compromised chondrogenic differentiation (Figure 2B). Remarkably, these phenotypic aberrations could be substantially rectified through *cxcl12a* mRNA microinjection (Figure 2B). Concomitantly, *cxcr4b* mutants exhibited no such craniofacial cartilage deformities (Figure 2B). Furthermore, gene expression assays targeting the extracellular primary cartilage matrix marker, *col2a1a*, undertaken at 72 hpf, ascertained a notable reduction in its expression levels exclusively in *cxcl12a* mutants, a condition reversible upon *cxcl12a* mRNA supplementation (Figure 2B).

To augment our understanding of *cxcl12a*'s involvement in craniofacial cartilaginous morphogenesis, an antisense *cxcl12a* MO targeting *cxcl12a* mRNA—a MO previously validated—was employed to attenuate *cxcl12a* expression (Gamba et al., 2010; Jiang et al., 2019). To assess the efficacy of this MO-mediated silencing, a plasmid harboring both the target sequence of the *cxcl12a* MO and eGFP was synthesized. MO efficiency was subsequently substantiated through the quantification of fluorescence intensity (Supplementary Figure S3A). Perturbations in *nanos3* expression were also evident following *cxcl12a* knockdown (Supplementary Figure S3B), corroborating both the specificity and efficacy of the *cxcl12a* MO. Consonant with observations in *cxcl12a* mutants, morphants displayed manifest aberrations in pharyngeal cartilage formation, defects that were ameliorable by *cxcl12a* mRNA injection (Supplementary Figure S2D). Collectively, Based on these data, these findings validate that the observed craniofacial cartilage malformations in either *cxcl12a* mutants and morphants emanate from *cxcl12a* transcriptomic attenuation, effectively obviating off-target effect of CRISPR/Cas9 effects.

Previously, the Cxcl12b-Cxcr4a axis has been delineated as requisite for CNCC migration and patterning during craniofacial ontogenesis (Olesnicky Killian et al., 2009). Given this, we postulated that *cxcl12a* and *cxcl12b* might exert synergistic functions in this developmental process. To interrogate this hypothesis, we deployed a previously characterized *cxcl12b* MO to suppress *cxcl12b* expression in *cxcl12a* mutants (Cha et al., 2012; Nguyen et al., 2014). The efficaciousness of *cxcl12b* MO was ascertained via fluorescence intensity metrics and *in situ* hybridization employing a *cxcl12b*-specific probe (Supplementary Figures S3A, C). Evaluation of cartilaginous elements in *cxcl12b* morphants revealed a pronounced diminution or outright absence of the ethmoid plate, coupled with trabecular fusion, corroborating prior literature consistent with previous report (Supplementary Figure S3D) (Olesnicky Killian et al., 2009). Remarkably, when *cxcl12b* MO was introduced into *cxcl12a* mutants, the resultant phenotypic distortions in pharyngeal cartilage were exacerbated; specifically, both Meckel’s cartilage and palatoquadrate were gravely malformed, and ceratobranchial cartilages were virtually obliterated (Figures 2A, B). Intriguingly, the simultaneous microinjection of *cxcl12a* and *cxcl12b* mRNA substantively restored craniofacial cartilage integrity in double-knockdown morphants (Figures 2A, B). Thus, our findings affirm that *cxcl12a* principally orchestrates craniofacial cartilage formation, whilst *cxcl12b* downregulation independently induces ethmoid plate atrophy and trabecular fusion.

### 3.3 *cxcl12a* has minor effect on CNCC migration

**FIGURE 3:**
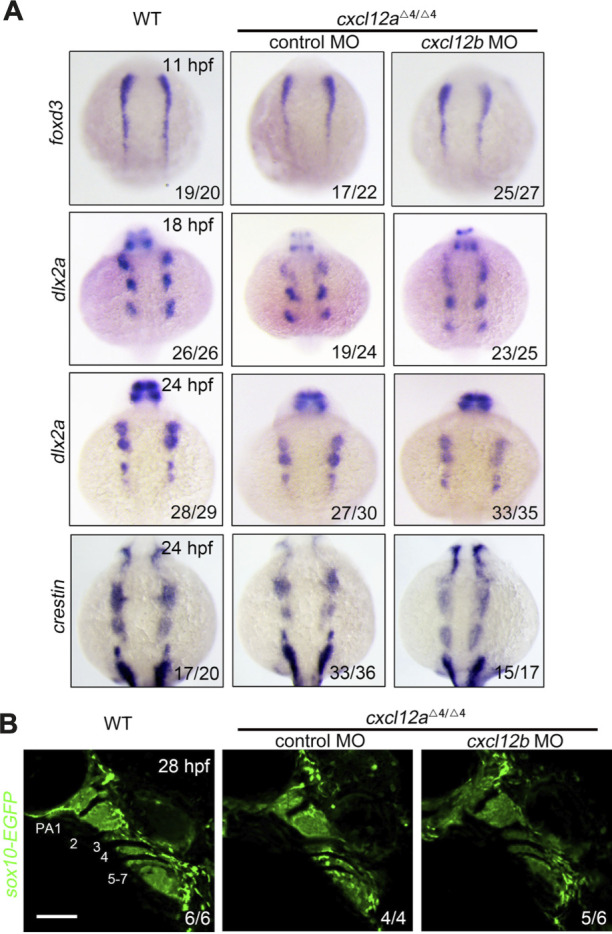
NCC specification and migration. **(A)** Comparative *in situ* hybridization analysis of wild type and *cxcl12a* mutant embryos administered either control MO or *cxcl12b* MO, harvested at indicated time points and probed for *foxd3*, *dlx2a* and *crestin*. **(B)** *In vivo* imaging of the pharyngeal compartment of wild type and *cxcl12a* mutants injected with either control MO or *cxcl12b* MO, set against a *Tg(sox10:EGFP)* reporter background. Pharyngeal arches are numerically annotated. PA denotes pharyngeal arch. Scale bars are set at 50 μm.

It is universally acknowledged that cranial neural crest cells (CNCCs) originate from the neural plate and subsequently undergo an epithelial-to-mesenchymal (ETM) transition. Following this transformation, CNCCs traverse toward pharyngeal arches and eventually differentiate into the chondrocytes constituting facial cartilage (Dash and Trainor, 2020). In order to elucidate the ramifications of *cxcl12a* deletion on CNCC activity, we ascertained the expression profiles of specific neural crest cell (NCC) markers across temporal stages. Remarkably, the expression of the neural crest cell specification marker *foxd3* remained congruent among control embryos, *cxcl12a* mutants and *cxcl12a* ^*cxcl12b*^ ^MO^ at the 5-somite stage (Figure 3). This uniformity in expressions trends indicates that *cxcl12a* is inconsequential to the induction and specification of the early neural crest cells.

To further scrutinize the impact on NCC migration, we employed *dlx2a* and *crestin* as migration markers. Within the time frame of 18–24 h post-fertilization (hpf) in control embryos, both *dlx2a* and *crestin* transcripts were profusely localized within the three neural crest cell groups. Nonetheless, only a marginal decrement in the expression levels of *dlx2a* and *crestin* was discernible in *cxcl12a* mutants at both 18 hpf and 24 hpf (Figure 3A). Conversely, injection of *cxcl12b* MO into *cxcl12a* mutants exacerbated the impediments to NCC migration (Figure 3A). *In vivo* imaging of the *Tg(sox10-EGFP)* line at 24 hpf corroborated that arch structures in control embryos and *cxcl12a* mutants were comparable, whereas the number of CNCCs in *cxcl12a* ^*cxcl12b*^ ^MO^ organisms was conspicuously reduced, aligning with prior studies (Figure 3B). Collectively, these findings substantiate the notion that the abrogation of *cxcl12a* exerts a minimal influence on CNCC migratory patterns.

### 3.4 *cxcl12a* is necessary pharyngeal arch patterning through promotes CNCC proliferation

**FIGURE 4:**
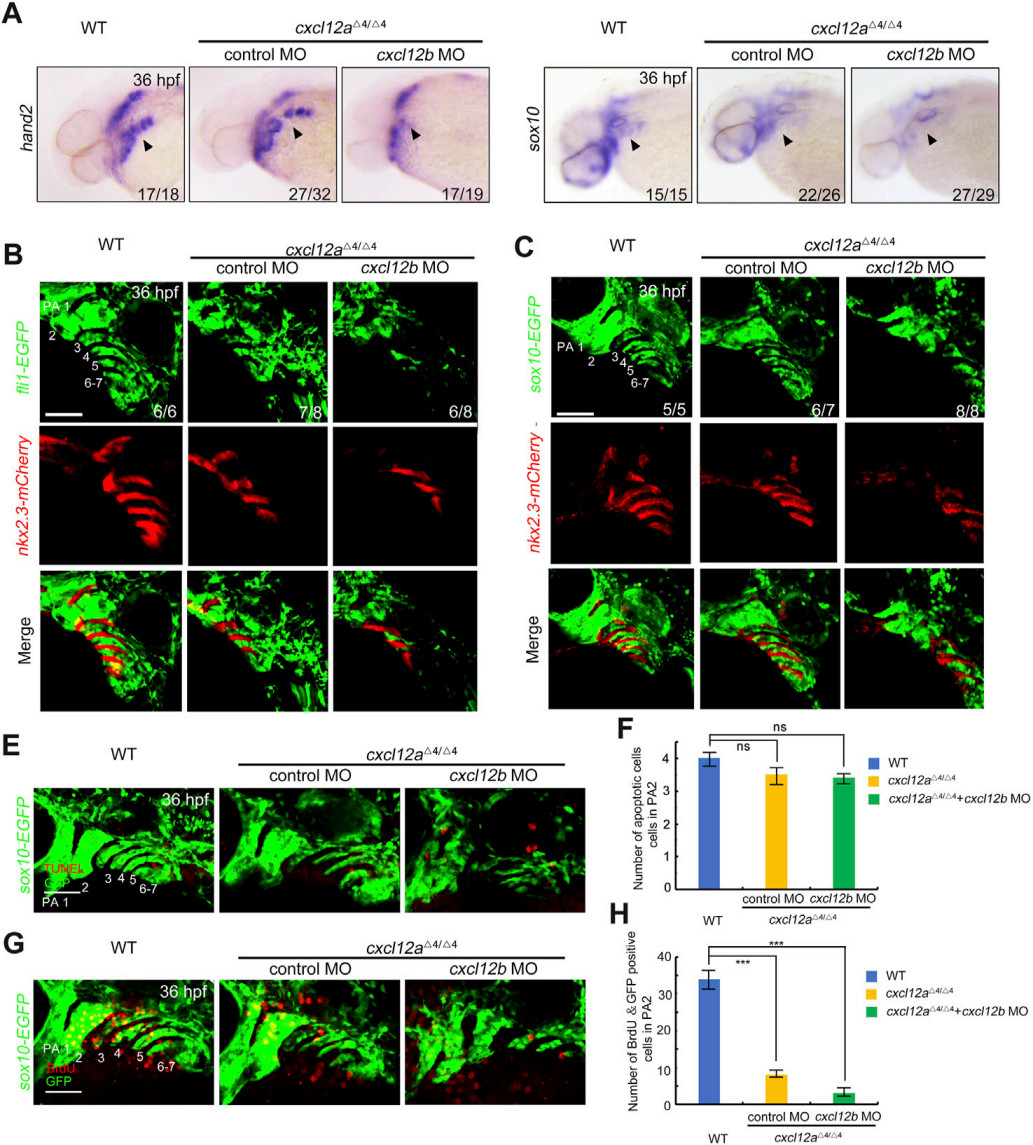
*Cxcl12a* function in CNCC proliferation after migration. **(A)** WT and *cxcl12a* mutant embryos subjected to control MO or *cxcl12b* MO administration were harvested at 36 hpf and interrogated via *in situ* hybridization employing *hand2* and *sox10* specific probes. Embryonic lateral views are presented with anterior orientation to the left. **(B–C)** *In vivo* live confocal micrographs of *Tg(nkx2.3:mCherry;fli1:GFP)* or *Tg(nkx2.3:mCherry;sox10:EGFP)* transgenic embryos injected with control MO, *cxcl12a* MO, or a combination of *cxcl12a* and *cxcl12b* MO at 24 hpf. Scale bar, 50 μm. **(E,F)** Apoptotic cell assessment in pharyngeal domains via TUNEL assays in *cxcl12a* mutants and wild-type siblings, injected with either control MO or *cxcl12b* MO against a *Tg(sox10:EGFP)* background. Pharyngeal arches (PA) are indicated. Scale bars represent 50 μm. Quantitative analysis of TUNEL-positive and GFP-positive cells within the pharyngeal arch was performed from nine independent embryos **(F)**. Error bars depict standard deviation (SD). NS signifies statistical non-significance as per Student’s *t*-test. **(G,H)** Bromodeoxyuridine (BrdU) incorporation assays reveal attenuated proliferative activity within CNCC populations in *cxcl12a* mutants subjected to control MO or *cxcl12b* MO treatment. Harvested embryos expressing *sox10:EGFP* were immunostained with anti-BrdU (red) and anti-GFP (green) antibodies. Pharyngeal regions were visualized through confocal microscopy **(G)**. Scale bars are set at 50 μm. Quantification of BrdU-positive and GFP-positive cells within the pharyngeal arch was achieved from nine independent embryos **(H)**. Error bars represent standard deviation (SD). Statistical significance denoted by ***, *p* < 0.001 as determined by Student’s *t*-test.

Extant literature delineates that the post-migratory CNCCs in the arches ultimately differentiate into cartilaginous chondrocytes (Hirata and Hall, 2000; Clouthier and Schilling, 2004), a process modulated by pharyngeal pouches serving as pivotal signaling centers (Piotrowski and Nusslein-Volhard, 2000; Couly et al., 2002; Crump et al., 2004). Given the localized expression of *cxcl12a* in these pharyngeal pouches post-CNCC migration (Figure 1C), we scrutinized the repercussions of *cxcl12a* depletion on these cells by assessing the expression profiles of *hand2* and *sox10* at 36 hpf. Notably, a quantitative decline in CNCCs was observed in both *cxcl12a* mutants and morphants in comparison to these control embryos, a reduction that could be successfully mitigated through the exogenous administration of *cxcl12a* mRNA (Figure 4A and Supplementary Figure S3E). To corroborate these observations, we employed quantitative reverse-transcription polymerase chain reaction (RT-qPCR) and whole-mount *in situ* hybridization (WISH) analyses, both of which yielded results in consonance with the initial assessments (Supplementary Figure S4). In line with these findings, comparable results were observed in *cxcl12a* morphants in *Tg(nkx2.3:mCherry;fli1:GFP)* or *Tg(nkx2.3:mCherry; sox10:GFP)* background, characterized by GFP expression in CNCCs at 36 hpf. These morphants exhibited disarrayed and abbreviated pharyngeal arch structures subsequent to *cxcl12a* knock-down (Figures 4B, C). Moreover, pharyngeal pouch patterning was exacerbated in *cxcl12a* ^*cxcl12b*^ ^MO^ mutants (Figures 4A–C).

To discern whether this observed phenotype could be attributed to alterations in CNCC apoptosis or proliferation, TUNEL staining and Bromodeoxyuridine (BrdU) incorporation assays were conducted on the *Tg(sox10:EGFP)* transgenic reporter line. Intriguingly, apoptosis levels in chondrogenic cells remained invariant between *cxcl12a* mutants and control embryos (Figures 4E, F). However, a pronounced diminution in the proliferation of BrdU^+^ CNCCs was detected following *cxcl12a* depletion (Figures 4G, H). Further compounding this effect, injection of *cxcl12b* MO into *cxcl12a* mutants culminated in even more severe pharyngeal arch malformations, attributable to a substantially diminished proliferative capacity of CNCCs (Figure 4 and Supplementary Figure S4).

Collectively, these empirical findings substantiate the indispensable role of *cxcl12a* in fostering CNCC proliferation subsequent to their migration.

### 3.5 Cxcl12a promotes pharyngeal pouch formation

**FIGURE 5:**
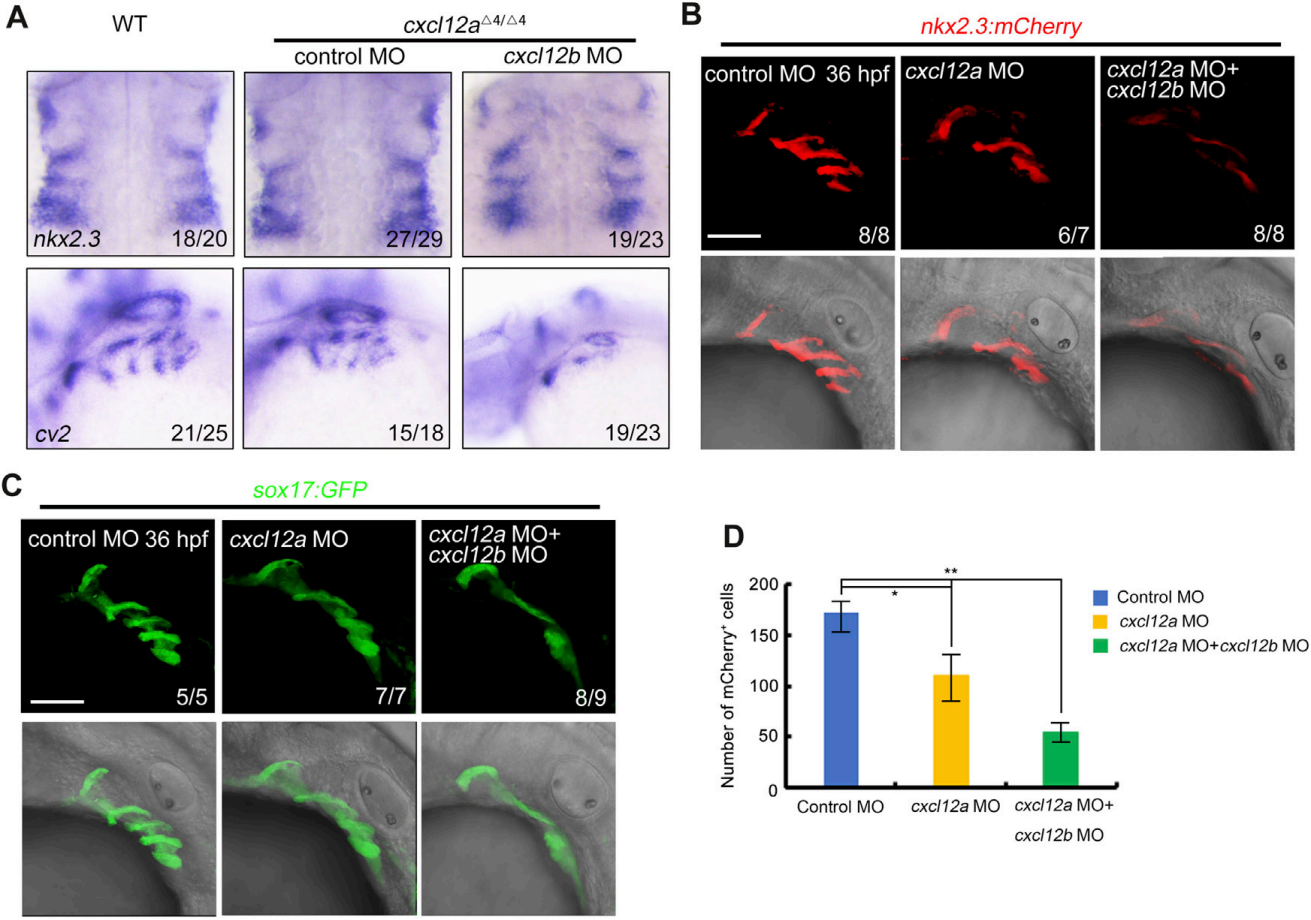
*cxcl12a* mutants exhibit reduced pharyngeal pouch cells. **(A)** Expression levels of *nkx2.3* and *cv2* expression were assayed in wild-type or *cxcl12a* mutants post-injection with either control MO or *cxcl12b* MO. **(B–C)** Confocal microscopy elucidates images showing pharyngeal pouch cellular architecture in wild-type and *cxcl12a,* or combined *cxcl12a* and *cxcl12b* knockdown embryos within a *Tg(nkx2.3:mCherry)* **(B)** or *Tg(sox17:GFP)* **(C)** background at 36 hpf. Scale bar, 50 μm. **(D)** Quantification of mCherry-positive cells within the *Tg(nkx2.3-mCherry)* transgenic line was executed from a sample size of nine embryos. Error bars are indicative of standard deviation (SD). Statistical significance denoted by *, *p* < 0.05; **, *p* < 0.01, as gauged by Student’s *t*-test.

Previous empirical evidence presented in Figures 4B, C elucidated that microinjection of *cxcl12a* MO in transgenic embryos with the *Tg(nkx2.3:mCherry;fli1:GFP)* or *Tg(nkx2.3:mCherry;sox10:EGFP)* background engendered a discernible diminution in mCherry-expressing pharyngeal pouches at 36 hpf. Given the co-localization of *cxcl12a* expression within the pharyngeal pouch, we conducted a meticulous examination of pharyngeal endoderm marker *nkx2.3* and *cv2* in both control and *cxcl12a* mutant embryos, either in the presence or absence of *cxcl12b* MO injections. Corroborating our earlier observations, the patterning of the pharyngeal pouches appeared perturbed in *cxcl12a* mutants consistent with our observation (Figure 5A).

To substantiate our preliminary findings, we deployed transgenic embryos featuring the *Tg(nkx2.3-mCherry)* and *Tg(sox17-GFP)* genetic backgrounds to serve as indicators of pharyngeal pouch cells. Our analysis revealed that the structural integrity of the pharyngeal pouch was compromised upon *cxcl12a* MO injection, manifesting as disorganized patterning (Figures 5B–D). Additionally, concomitant microinjection of *cxcl12a* MO with *cxcl12b* MO resulted in exacerbated perturbations in pharyngeal pouch morphology (Figures 5B–D). In summation, these empirical observations validate the cooperative functionality of *cxcl12a* and *cxcl12b* in orchestrating the formation of the pharyngeal pouch.

## 4 Discussion

In the present investigation, we delineated that *cxcl12a* exhibits a spatially restricted expression within the pharyngeal pouches, and its abrogation precipitates anomalies in pharyngeal cartilage formation, attributable to attenuated post-migratory cranial neural crest cell (CNCC) proliferation. Concurrent knockdown *cxcl12b* in *cxcl12a* mutations or morpholinos intensifies the severity of defects in both craniofacial cartilage and pharyngeal pouch formation, thereby substantiating the hypothesis that *cxcl12a* and *cxcl12b* have synergistic influence on pharyngeal cartilage morphogenesis and pouch development. Collectively, these findings elucidate the pivotal role of *cxcl12a* in the proliferative dynamics of post-migratory CNCCs.

The specificity of *cxcl12a* expression to the pouch endoderm (Figure 1) contrasts with *cxcl12b*'s broader expression profile, which encompasses both the pharyngeal pouch and arch mesenchyme (Olesnicky Killian et al., 2009). These differential expression patterns engender the supposition that *cxcl12a* performs a cell-autonomous function in the formation of the pharyngeal pouch and a cell non-autonomous function in the pharyngeal arch, whereas *cxcl12b* exhibits a dual role, influencing both arch and pouch development through cell-autonomous mechanisms. The veracity of this conjecture could be elucidated via tissue-specific ablation of *cxcl12a*/*b*, necessitating further experimental validation.

In this study, *cxcr4b* null mutants were generated through the CRISPR/Cas9-mediated gene editing. Notably, these mutants exhibited no conspicuous anomalies, corroborating prior reports (Liu et al., 2022). CXCR7, a recently characterized G-protein coupled receptor, demonstrates affinity for two chemokine ligands-CXCL11 and CXCL12—with a pronounced binding affinity for CXCL12 (Naumann et al., 2010; Sanchez-Martin et al., 2013). Given these antecedents, we postulate that CXCR7 could potentially interact with Cxcl12a to facilitate CNCC proliferation in the absence of Cxcr4b, albeit this hypothesis warrants further empirical scrutiny.

Previous literature has indicated that Cxcl12a augments chondrocyte proliferation via the ERK1/2-cyclin D1 signaling pathway, a finding concordant with our results (Kim et al., 2015). Nonetheless, the downstream signaling cascade implicated in *cxcl12a*-mediated CNCC proliferation remains elusive and calls for further investigative efforts. Previous reports have demonstrated that Cxcr4 and Sdf1 are genetically downstream of Tbx1 in pharyngeal neural crest cell ontogeny, and a decrement in CXCR4 signaling has been linked to impaired CNCC migration in murine and avian models (Escot et al., 2016; Page et al., 2018). In zebrafish embryos, *cxcl12b* operates downstream of *dmrt2b* within endodermal pouches and modulates CNC cell compaction within the pharyngeal arches (Li et al., 2018). Nevertheless, morphants of *cxcl12b* and *cxcr4a* exhibit only modest defects in CNCC migration and craniofacial cartilage development (Olesnicky Killian et al., 2009). We posit that while Cxcl12b/Cxcr4a predominantly modulates CNCC migration, *cxcl12a* exerts a crucial influence on CNCC proliferation post-migration, an assertion that merits comprehensive molecular elucidation in future studies.

## 5 Conclusion

In summation, the ontogenetic paradigms that govern craniofacial cartilage morphogenesis and NCC development are intricate, encompassing a multifaceted interplay of signaling cascades and cellular interactions across diverse cell types. The current investigation underscores the quintessential function of the zebrafish-specific chemokine cxcl12a in the orchestration of craniofacial cartilage formation and post-migratory CNCC proliferation. Consequently, this study illuminates a heretofore uncharacterized molecular mechanism that modulates craniofacial cartilage development through the promotion of CNCC proliferation in the post-migratory phase, mediated by the pharyngeal pouch-localized chemokine *cxcl12a*.

## Data Availability

The datasets presented in this study can be found in online repositories. The names of the repository/repositories and accession number(s) can be found in the article/Supplementary Material.
