# Supplementary material for: cxcl12a plays an essential role in pharyngeal cartilage development
Source: Front Cell Dev Biol. 2023 Oct 4;11:1243265. doi: 10.3389/fcell.2023.1243265 (PMC10582265; doi:10.3389/fcell.2023.1243265)
Supplement: Supplementary file 1 [file DataSheet1.PDF]

Supplementary Material

cxcl12a plays an essential role in pharyngeal cartilage development

Authors: Zhaohui Wei, Jingwen Liu*

*** Correspondence: 1205099802@qq.com**

Supporting information

Supplementary Figure 1

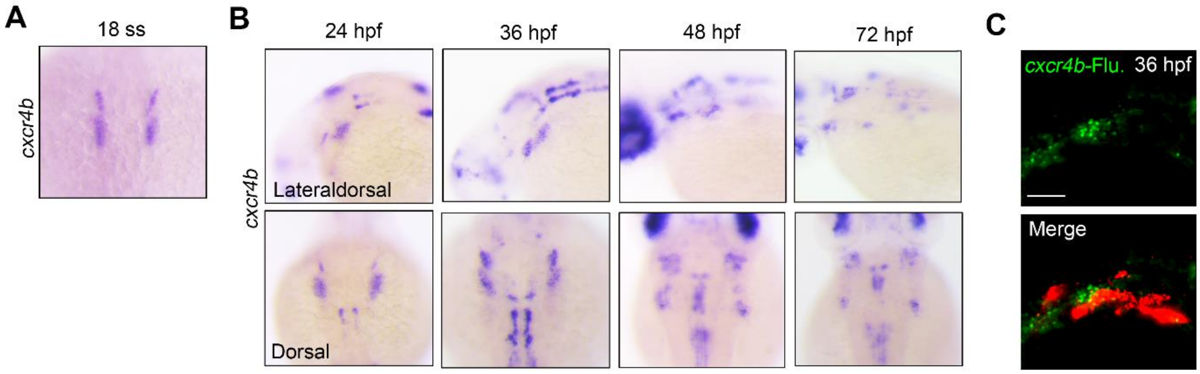

Supplementary Figure 1. The expression of *cxcr4b* in pharyngeal region. (A) *In situ* hybridization was employed to elucidate the expression profile of *cxcr4b* at 18-somite stage， with the animal pole oriented at the apex.. (B) Longitudinal assessment of *cxcr4b* expression from 24 hpf to 72 hpf is presented in a latero-dorsal, with the animal pole situated at the summit in the left frame.(C) Dual-color fluorescence *in situ* hybridization was conducted on *Tg(nkx2.3:mCherry)* embryos to further specify the localization of *cxcr4b* . Scale bar corresponds to 50 µm.

Supplementary Figure 2

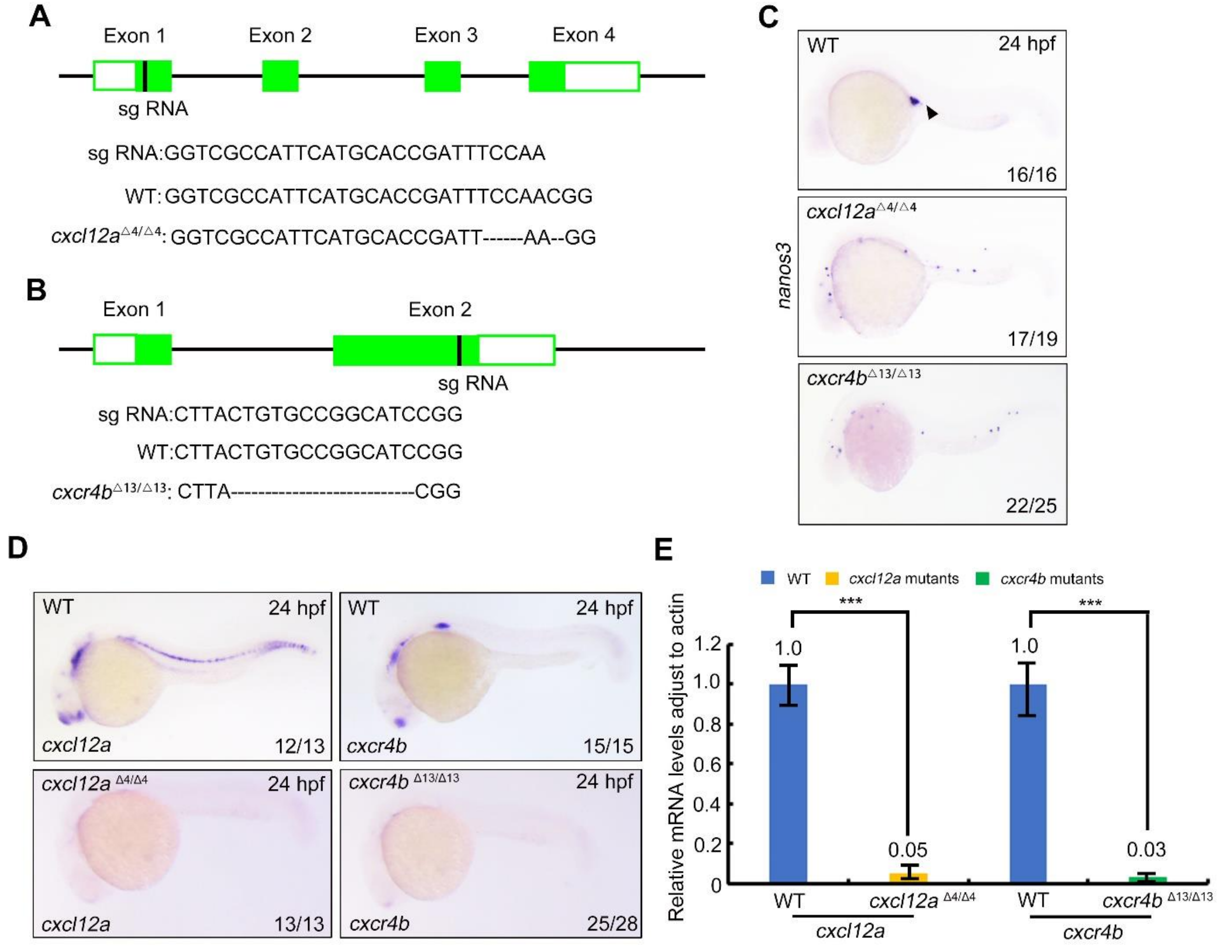

Supplementary Figure 2. The validation of *cxcl12a* and *cxcl12b* mutants. (A-B) Generation of targeted mutants for *cxcl12a* and *cxcr4b* was executed utilizing the CRISPR/Cas9 genomic editing system. The *cxcl12a* mutant harbors a quad-base deletion, leading to the synthesis of a truncated protein isoform (A). Concurrently, the *cxcr4b* mutants possess a 13-base pair deletion, which similarly culminates in a truncated transmembrane domain protein (B). (C) Expression of *nanos3* was assessed in wild-type embryos, as well as in *cxcl12a*$^{\triangle 4/\triangle 4}$ and *cxcr4b*$^{\triangle 13/\triangle 13}$ mutants at 24 hpf. The vertex of the triangle demarcates the locale of primordial germ cells. (D) Spatial expression patterns of *cxcl12a* and *cxcr4b* were investigated via in situ hybridization techniques at 24 hpf. (E)

Quantitative reverse transcription polymerase chain reaction (RT-qPCR) was utilized to examine the relative expression levels of *cxcl12a* and *cxcr4b* in wild-type, *cxcl12a*, and *cxcr4b* mutant embryos. β-actin served as an internal calibration standard. Data are represented as the mean ± standard deviation (SD) from three biologically independent replicates. Total RNA for each experimental group was pooled from an ensemble of 30 embryos. Statistical significance was assessed by Student's t-test, with ***, $p < 0.001$ indicating extreme significance.

Supplementary Figure 3

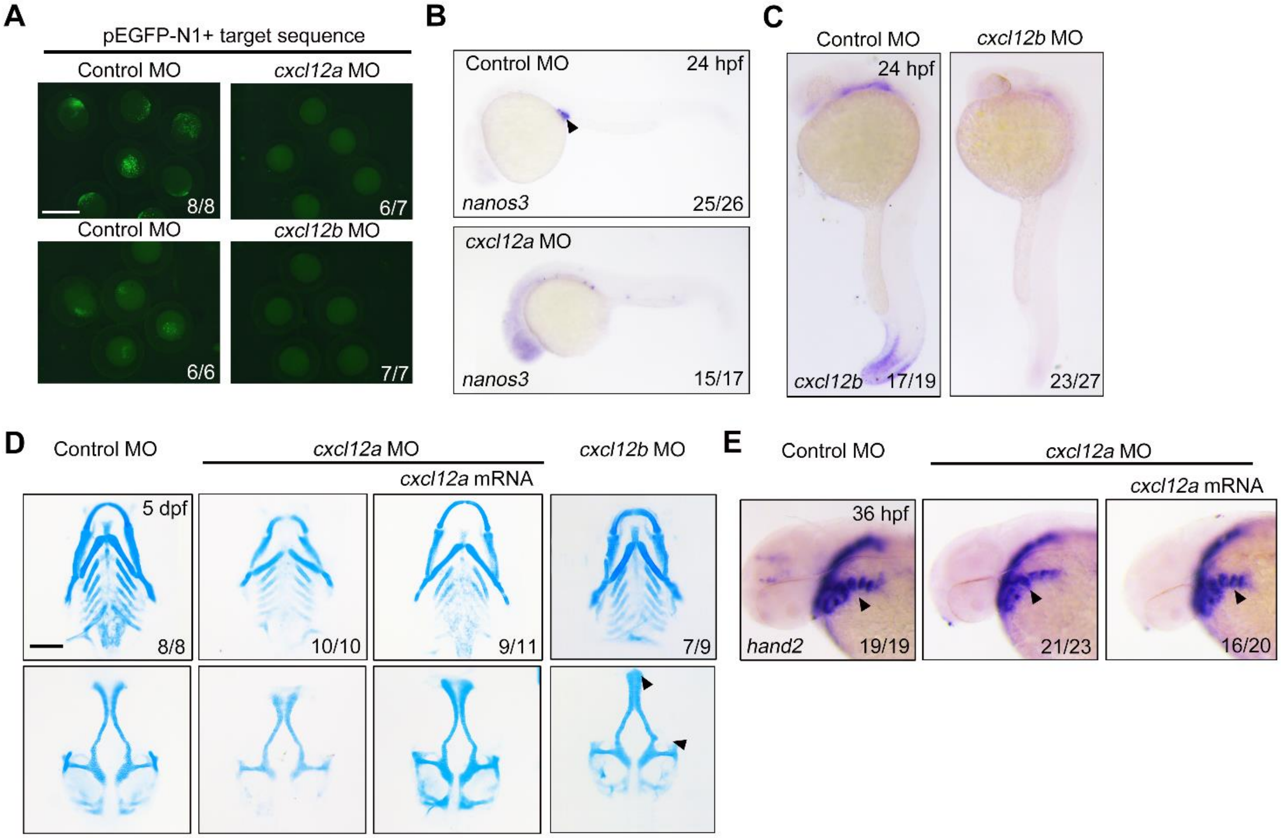

Supplementary Figure 3. *cxcl12a* and *cxcl12b* MO verification and morphant developmental defects. (A) Reporter mRNA comprising the 5' untranslated region and a portion of the coding sequence of enhanced green fluorescent protein (EGFP-5'UTR-part of CDS) was injected at a concentration of 100 pg into single-cell embryos either alone or in conjunction with control morpholino (MO), *cxcl12a* MO, or *cxcl12b* MO. Subsequent evaluation of GFP fluorescence was executed at 8 hours post-fertilization (hpf). Scale bar signifies 200 mm. (B) For *in situ* hybridization analyses against the *nanos3* probe, wild-type embryos were subjected to injections of either control MO (cMO) or *cxcl12a* MO at 24 hpf. The apex of the triangle demarcates the spatial positioning of primordial germ cells. (C) Temporal and spatial expression trajectories of *cxcl12b* in *cxcl12b* morphant embryos relative to their wild-type siblings were elucidated through *in situ* hybridization at

designated developmental stages. (D) Alcian blue staining protocols were implemented on embryos receiving injections of control MO, *cxcl12a* MO, and optionally, *cxcl12a* mRNA. Scale bars represent 100 mm. (E) *hand2* gene expression profiles in *cxcl12a* mutant embryos, both with and without supplemental *cxcl12a* mRNA, were examined via *in situ* hybridization.

Supplementary Figure 4

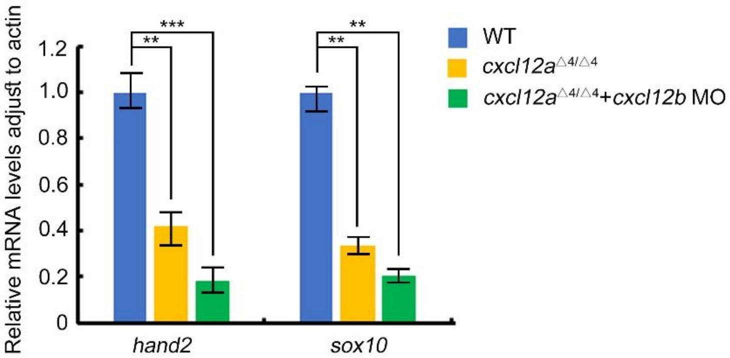

Supplementary Figure 4. RT-qPCR analysis of the mRNA levels of *hand2* and *sox10.* Relative transcriptional abundance was ascertained employing the $2^{-\Delta\Delta Ct}$ computational methodology. β-actin functioned as an endogenous normalization control. Data are articulated as the mean ± standard deviation (SD) derived from three biologically independent repetitions. Collective RNA samples for each experimental cohort were isolated from an assemblage of 30 embryos. Statistical inference was achieved through Student's t-test; ** denotes $p < 0.01$, and *** indicates $p < 0.001$.
